# Mining algorithm of accumulation sequence of unbalanced data based on probability matrix decomposition

**DOI:** 10.1371/journal.pone.0288140

**Published:** 2023-07-07

**Authors:** Shaoxia Mou, Heming Zhang

**Affiliations:** 1 University of Perpetual Help System Dalta, Graduate School Eternal University, Las Piñas, Philippines; 2 Binzhou Medical University Hospital, Binzhou, Shandong, China; TU Wien: Technische Universitat Wien, AUSTRIA

## Abstract

Due to the inherent characteristics of accumulation sequence of unbalanced data, the mining results of this kind of data are often affected by a large number of categories, resulting in the decline of mining performance. To solve the above problems, the performance of data cumulative sequence mining is optimized. The algorithm for mining cumulative sequence of unbalanced data based on probability matrix decomposition is studied. The natural nearest neighbor of a few samples in the unbalanced data cumulative sequence is determined, and the few samples in the unbalanced data cumulative sequence are clustered according to the natural nearest neighbor relationship. In the same cluster, new samples are generated from the core points of dense regions and non core points of sparse regions, and then new samples are added to the original data accumulation sequence to balance the data accumulation sequence. The probability matrix decomposition method is used to generate two random number matrices with Gaussian distribution in the cumulative sequence of balanced data, and the linear combination of low dimensional eigenvectors is used to explain the preference of specific users for the data sequence; At the same time, from a global perspective, the AdaBoost idea is used to adaptively adjust the sample weight and optimize the probability matrix decomposition algorithm. Experimental results show that the algorithm can effectively generate new samples, improve the imbalance of data accumulation sequence, and obtain more accurate mining results. Optimizing global errors as well as more efficient single-sample errors. When the decomposition dimension is 5, the minimum RMSE is obtained. The proposed algorithm has good classification performance for the cumulative sequence of balanced data, and the average ranking of index F value, G mean and AUC is the best.

## Introduction

In modern technology and business applications, people are often faced with various types of data sets. Many of these data sets are lopsided, with some categories having very small sample sizes and others having very large sample sizes. This situation is very common in machine learning and data mining, which will not only affect the prediction results of the model, but also lead to the failure of the data mining algorithm to explore the potential rules and information. Therefore, processing unbalanced data sets is a very important problem in the field of data mining and machine learning.

The methods to deal with unbalanced data sets include but are not limited to: using different evaluation indicators, oversampling or under sampling, integrated learning, cost-sensitive learning, transfer learning, etc., and the popular meta-learning methods in recent years can also be applied to unbalanced data sets. Processing unbalanced data sets can improve the performance and generalization ability of the model and find more valuable information, so it has high practical application value. In the past decades, the rapid development of global information technology has led to the emergence of powerful computers, data collection devices and storage devices. With these devices, a large amount of data information can be collected for transaction management, information retrieval and data analysis [1]. Although the amount of data collected is very large, the data useful to people is often very limited, usually only a small part of the total data. This kind of data accumulation sequence in which the number of samples of a certain class is significantly less than that of other classes is called accumulation sequence of unbalanced data [2]. In unbalanced data, there is a large difference in the number of samples in each category. Non balanced data classification, that is, the number of one type of samples in the data set is far greater than that of other types. The type of samples that account for the majority is called the majority type, while the other type that only accounts for a small part is called the minority type. The cumulative sequence of unbalanced data is widely used in credit risk assessment, fraud detection, disease diagnosis, weather forecast and other application fields. The classification of such unbalanced data has one thing in common, that is, the focus is on a few types of information. For non-equilibrium data sets, an intuitive feature is data scarcity, which is obviously one of the reasons for the sharp decline in classification performance. If it is misclassified, it will bring significant losses. The classification problem of accumulation sequence of unbalanced data exists in people’s real life and industrial production [3]. For example, the number of fleeing customers looking for telecom operators is generally far less than that of non fleeing customers; Using detection data to diagnose patients’ diseases, such as cancer, the probability of people suffering from cancer is very low, so cancer patients are far less than healthy people; Others include locating oil wells from satellite images, learning the pronunciation of words, automatic text classification, and distinguishing malicious harassing calls. In these applications, people are mainly concerned about the minority classes in the data accumulation sequence, and the cost of misclassification of these minority classes is very large [4]. Taking the credit evaluation problem as an example, there are relatively few samples of customers with poor credit. When mining them, the traditional mining algorithm focuses on the overall mining accuracy, which is easy to cause miscalculations of a few categories, resulting in a high error rate that a few customers with poor credit are misclassified as good customers. If loans are allocated to them, it will cause great capital losses to the enterprise. In cumulative sequence mining, the length of accumulated sequences of different categories may vary greatly. More common categories may form longer accumulation sequences, and correspondingly, less common categories may form shorter accumulation sequences. As a result, when the algorithm is mining, the more common patterns will be more easily excavated, while the less common patterns will be easily ignored. Moreover, the length of the excavated sequence may be much larger than the length of the real sequence. All these problems will affect the accuracy of the mining results. If the scale of raw materials is large, the data accumulation sequence mining algorithm may need to support large-scale data processing, and the efficiency and scalability of the algorithm become challenges. However, if the scale of the original material is too small, the cumulative sequence mined may not represent the whole data set, which will lead to the inaccuracy of the mining results. Therefore, in practical applications, the mining of accumulation sequence of unbalanced data has been paid more and more attention by the academic circles of data mining and machine learning, and has become one of the hot issues in the field of data mining and machine learning [5]. In 2000, the American Association of Artificial Intelligence (AAAI) and the 2003 International Conference on Machine Learning (ICML) held a symposium on learning unbalanced data. In 2004, the American Computer Society (ACM) published a newsletter on this topic. Therefore, the mining of accumulation sequence of unbalanced data is very important for data application, and it is urgent to study an effective mining method for accumulation sequence of unbalanced data.

In order to solve the problem of sparsity in accumulation sequence of unbalanced data and effectively realize the mining of accumulation sequence of unbalanced data, domestic and foreign scholars have done a lot of research. In reference [6], El-Saeiti and Khalil et al. had made in-depth research on the H-likelihood estimation method of accumulation sequence of unbalanced data. However, although random replication of a few samples in this method could quickly and directly increase the number of samples, the actual effect was not ideal and it was easy to produce over fitting. In reference [7], Tang and Cai et al. conducted in-depth research on mixed feature selection for diagnosis of unbalanced cancer data. However, although this method improved the classification performance of a few samples to a certain extent, it did not take into account the imbalance within a few samples and was easy to introduce noise points. In reference [8], Carrillo-Alarcón and Morales-Rosales conducted in-depth research on parameter estimation in unbalanced data classification. The time complexity and spatial complexity of the research methods were particularly high.

Based on the above problems, this paper studies the mining algorithm for accumulation sequence of unbalanced data under probability matrix decomposition to improve the mining performance of accumulation sequence of unbalanced data.

## Materials and methods

### Accumulation sequence of unbalanced data preprocessing

In order to solve the problems of imbalance in the minority samples and noise and overlap in the newly generated samples in the accumulation sequence of unbalanced data [9], an unbalanced data processing method is designed, which combines the natural nearest neighbor relationship of the minority samples and the internal cluster to which they belong. The proposed method first calculates the natural nearest neighbor of minority samples according to the definition of natural nearest neighbor, obtains the core points in the densely distributed area of samples, then clusters the minority samples based on the natural nearest neighbor relationship, and finally uses the core points and non core points in the same category to synthesize new samples, and adds them to the original accumulation sequence of unbalanced data, so as to balance the accumulation sequence of unbalanced data.

#### Minority clustering in accumulation sequence of unbalanced data based on natural nearest neighbor

The density clustering of minority samples in the accumulation sequence of unbalanced data based on the natural nearest neighbor relationship mainly includes three steps [10]: firstly, the core point is determined according to the natural nearest neighbor relationship of all minority samples in the accumulation sequence of unbalanced data; the self-neighborhood radius of the core point is calculated, to determine the core point directly connected with its density, and cluster the core points according to the process of density clustering [11], so as to classify the two core points within their own neighborhood radius values into the same category; According to the nearest neighbor relationship between non core points and core points, non core points are classified into the existing classification.

The implementation process of clustering algorithm based on natural nearest neighbor for minority classes in accumulation sequence of unbalanced data is as follows.

Input: natural eigenvalue *s*_*k*_ of a few samples in the accumulation sequence of unbalanced data, natural nearest neighbor *N*_*N*_(*x*_*i*_) and natural nearest neighbor *N*_*B*_(*x*_*i*_) of sample point *x*_*i*_.

Output: core point set *C*_*s*_ of minority samples, non core point set *N*_*Cs*_, number of clusters *G* and sample set *C*_*G*_ of various clusters.

Step 1: determine *C*_*s*_ and *N*_*Cs*_ from *s*_*k*_, *N*_*N*_(*x*_*i*_) and *N*_*B*_(*x*_*i*_) according to definition clustering algorithm.

Step 2: cluster *x*_*i*_, *x*_*i*_∈*C*_*s*_.

Step 2.1: calculate the neighborhood radius *ϕ*_*i*_ of *x*_*i*_ and the core point set *D*_*i*_ of which the direct density can be reached.

Step 2.2: using *ϕ*_*i*_ and *D*_*i*_ and referring to the density clustering process, classify all density connected core points into the same cluster to obtain *C*_*G*_ and *G*.

Step 3: divide *x*_*j*_ into *G* classes, *x*_*j*_∈*N*_*Cs*_.

Step 3.1: calculate *ϕ*_*j*_ for *x*_*j*_.

Step 3.2: get all core points that are natural neighbors to *x*_*j*_ and within the radius of each other’s neighborhood.

Step 3.3: determine the core point *x*_*i*_ closest to *x*_*j*_, and add *x*_*j*_ to the cluster where *x*_*i*_ is located; If there is no such core point, mark *x*_*j*_ as a noise point.

In the above process, new samples are generated by core points and non core points belonging to the same cluster. Noise points do not participate in the synthesis of sample points, which can avoid the introduction of new noise points.

#### Oversampling based on natural nearest neighbor

After completing the clustering operation for a few samples in the accumulation sequence of unbalanced data, it can determine the number of samples to be synthesized for each class according to the proportion of non core points in each class. The more the non core points in the class are, the more new samples to be synthesized are. The reason is that the non core points are the samples whose number of natural nearest neighbors is less than the natural eigenvalue [12]. The distribution of samples around them is relatively sparse. More new samples are synthesized inside them, which can avoid the excessive concentration of new samples in densely distributed areas and reduce the overlap of composite samples. The specific steps of oversampling based on natural nearest neighbors are as follows.

Input: accumulation sequence of unbalanced data *S*.

Output: minority class sample set *S*_*g*_ generated by oversampling.

Step 1: search the natural nearest neighbor of a few samples [13] to obtain *s*_*k*_, *N*_*N*_(*x*_*i*_) and *N*_*B*_(*x*_*i*_).

Step 2: cluster a few samples to get *G*, *C*_*K*_, *C*_*s*_ and *N*_*Cs*_.

Step 3: number of newly synthesized samples *N* = number of samples of most classes—number of samples of few classes.

Step 4: calculate the proportion *p*_*i*_ of non core points in class *C*_*i*_, and determine the number of samples $N_{i}=(\frac{p_{i}}{\sum_{j=1}^{k}p_{i}})\times N$ to be synthesized for class *C*_*i*_ according to the proportion.

Step 5: randomly select non core point *x* and core point *y* in class *C*_*i*_ to synthesize new samples, *i* = 1,2,⋯,*G*. *z* = *x*+*a*×(*y*−*x*), *a* is a random number in the range of [0,1], add *z* to the set *S*_*g*_ and repeat this step until the required number of samples is obtained for each category.

*S*_*g*_ is the composite sample set of a few classes.

Through the above series of processes, new minority samples are synthesized and added to the original data accumulation sequence, so as to balance the data accumulation sequence.

### Mining of accumulation sequence of unbalanced data

#### Mining of accumulation sequence of unbalanced data based on probability matrix decomposition

Probabilistic Matrix Factorization (PMF) is a common recommendation system algorithm that uses a user-item rating matrix to recommend items that users might be interested in. In PMF, the original score matrix is decomposed to map both users and items into a potentially low-dimensional space, and then the score matrix is recalculated until its error from the original score matrix is minimal. Probability matrix decomposition is to map some potential information of users and projects (accumulation sequence of unbalanced data after balanced processing, hereinafter referred to as data sequence) to low dimensional feature space [14] from the perspective of probability, and then use the linear combination of low dimensional feature vectors to explain the preference of specific users for data sequences. Given the scoring matrix *R*_*m*×*n*_ of user data series, MATLAB is used to generate a random number matrix *H*_*f*×*m*_ with mean value of 0, variance of $\delta_{H}^{2}$ and a random number matrix *Q*_*f*×*n*_ with variance of $\delta_{Q}^{2}$, where *f* is the decomposition dimension, *H*_*f*×*m*_ is defined as the user’s *f*-dimensional characteristic matrix, *Q*_*f*×*n*_ is the *f*-dimensional characteristic matrix of data series, and its column vectors *H*_*i*_ and *Q*_*j*_ represent the corresponding potential characteristic vectors respectively, generally $R_{m\times n}\neq H_{f\times m}^{T}\times Q_{f\times n}$. After learning the training set, *H*_*f*×*m*_ and *Q*_*f*×*n*_ are constantly updated, to make $H_{f\times m}^{T}\times Q_{f\times n}\to R_{m\times n}$.

Assuming that the error between the real score *R*_*ij*_ and the predicted score ${\hat{R}}_{ij}$ conforms to the Gaussian distribution [15] with a mean value of 0 and a variance of $\delta_{R}^{2}$, there is a probability distribution: $p(R_{ij}-H_{i}^{T}Q_{j}|0,\delta_{R}^{2})$, and there is $p(R_{ij}|H_{i}^{T}Q_{j},\delta_{R}^{2})$ through translation, then the conditional probability of the scoring matrix *R* is as shown in Eq (1):

$$p\left(R|H,Q,\delta_{R}^{2}\right)=\prod_{i=1}^{m}\prod_{j=1}^{n}N^{I_{ij}}\times {\left(R_{ij}|H_{i}^{T}Q_{j},\delta_{R}^{2}\right)}^{I_{ij}}$$ (1)

Where, *I*_*ij*_ is the indicator function, *I*_*ij*_ value of 1 indicates that user i has scored the data sequence j, and *I*_*ij*_ value of 0 indicates that user i has not scored the data sequence j. *N* is the actual error, *δ*_*R*_ is the forecast error.

Since *H* and *Q* are independent of each other and satisfy the Gaussian distribution with mean value of 0 and variance of $\delta_{H}^{2}$ and $\delta_{Q}^{2}$ respectively, there are Eqs (2) and (3):

$$P\left(H|\delta_{H}^{2}\right)=\prod_{i=1}^{m}NH_{i}^{}|0,N\delta_{H}^{2}I$$ (2)

$$P\left(Q|\delta_{Q}^{2}\right)=\prod_{j=1}^{m}NQ_{i}^{}|0,N\delta_{Q}^{2}I$$ (3)

Where, *I* is the function value. The joint probability distribution of *H* and *Q* can be obtained from Eqs (1), (2) and (3):

$$\begin{array}{l} p(H,Q|R,\delta_{R}^{2},\delta_{H}^{2},\delta_{Q}^{2})={\prod_{i=1}^{m}\prod_{j=1}^{n}\left(NR_{ij}|H_{i}^{T}Q_{j},N\delta_{R}^{2}\right)}^{I_{ij}}\times \\ \;{}_{}{}_{}\prod_{i=1}^{m}NH_{i}^{}|0,N\delta_{H}^{2}I\times \prod_{j=1}^{n}NQ_{i}^{}|0,N\delta_{Q}^{2}I \end{array}$$ (4)

Taking logarithm of probability distribution of *U* and *V* can get:

$$\begin{array}{l} \ln p(H,Q|R,\delta_{R}^{2},\delta_{H}^{2},\delta_{Q}^{2})=-\frac{\prod_{i=1}^{m}\prod_{j=1}^{n}I_{ij}{\left(R_{ij}-H_{i}^{T}Q_{j}\right)}^{2}}{2\delta_{R}^{2}}-\frac{\sum_{i=1}^{m}H_{i}^{T}Q_{j}}{2\delta_{H}^{2}}-\frac{\sum_{j=1}^{n}H_{j}^{T}Q_{j}}{2\delta_{Q}^{2}} \end{array}$$ (5)

The maximum value of Eq (5) can be equivalently replaced by the minimum value of the error function with regularization parameters [16], as shown in Eq (6):

$$E_{\min}=\frac{\sum_{i=1}^{m}\sum_{j=1}^{n}I_{ij}{\left(R_{ij}-H_{i}^{}Q_{j}\right)}^{2}}{2}+\frac{\lambda H(\sum_{i=1}^{m}{\left\|H_{i}^{}\right\|}^{2}+\frac{\lambda H}{2}\sum_{j=1}^{n}{\left\|Q_{j}^{}\right\|}^{2})}{2}$$ (6)

Where, $\lambda_{H}=\frac{\delta_{R}^{2}}{\delta_{H}^{2}}$, $\lambda_{Q}=\frac{\delta_{R}^{2}}{\delta_{Q}^{2}}$, and $\delta_{H}^{2}=\delta_{Q}^{2}$, the objective function can be described by Eq (7):

$$E_{\min}=\frac{\sum_{i=1}^{m}\sum_{j=1}^{n}I_{ij}{\left(R_{ij}-H_{i}^{}Q_{j}\right)}^{2}}{2}+\frac{\lambda (\sum_{i=1}^{m}{\left\|H_{i}^{}\right\|}^{2}+\sum_{j=1}^{n}{\left\|Q_{j}^{}\right\|}^{2})}{2}$$ (7)

The relationship between the regularization parameter *λ* and $\delta_{R}^{2}$, $\delta_{H}^{2}$, $\delta_{Q}^{2}$ is obtained.

In order to solve the objective function, the random gradient descent method is used. This algorithm finds the direction in which the parameters of the objective function decline fastest by deriving the parameters [17], and makes the variables move along this direction until they move to the minimum point.

By deriving from Eq (7), it can be found that at each iteration, the updated equations of *H*_*i*_ and *Q*_*j*_ become:

$$e=R_{ij}-H_{i}^{T}Q_{j}$$ (8)

$$H_{i}^{}\leftarrow H_{i}^{}+\alpha (e\times Q_{j})-\alpha (\lambda \times H_{i}^{})$$ (9)

$$Q_{j}\leftarrow Q_{j}+\alpha (e\times H_{I})-\alpha (\lambda \times Q_{j}^{})$$ (10)

Where *α* is the learning rate of random gradient descent.

In addition, in order to improve the recommendation efficiency of the algorithm, the batch processing module [18] is added. For 90000 training data in the experiment, it is divided into 9 batches and 10000 data are processed each time, which greatly reduces the amount of calculation of model training and the instability of model convergence caused by the calculation of each training data.

The data sequence mining algorithm based on probability matrix decomposition is as follows:

Input: training set *train*_*vce*_, test set *probe*_*vce*_.

Output: mining result *pred*_*out*_, square root error *REST*.

1. Set the regularization parameters, the maximum number of iterations maxepoch, and decompose the dimension *feat*;
2. Generate two standard normal distribution matrices: data sequence (1682) *feat* and number of users (943) *feat*;
3. Iteration times epoch < maxepoch;
4. Batch processing is adopted, which is divided into 9 batches, 10000 scoring records are processed each time, and the batch processing times are batch < 9;
5. The loss function *p* is calculated, and the two matrices of (2) are continuously updated according to the negative gradient direction;
6. End.

Through the above process of accumulation sequence of unbalanced data preprocessing and probability matrix decomposition, accumulation sequence of unbalanced data mining can be realized. The unbalanced data accumulation sequence is partially coded as follows:

#include <iostream>#include <cstring>#include <map>using namespace std;const int N = 1e6+10;#define int long longint n, maxn, minn, top1, top2, ans;int a[N], Lb[N], Rb[N], Ls[N], Rs[N];int st1[N], st2[N];signed main(){

  cin >> n;

  for (int i = 1; i < = n; i++) cin >> a[i];

  top1 = 0, top2 = 0;

  for (int i = 1; i < = n; i++)

  {

 while (top1 && a[st1[top1]] > a[i]) top1—;

 while (top2 && a[st2[top2]] < a[i]) top2—;

 if (!top1) Ls[i] = 0;

 else Ls[i] = st1[top1];

 if (!top2) Lb[i] = 0;

 else Lb[i] = st2[top2];

 st1[++top1] = i;

 st2[++top2] = i;

  }

  memset(st1, 0, sizeof(st1));

  memset(st2, 0, sizeof(st2));

  top1 = 0, top2 = 0;

  for (int i = n; i > = 1; i—)

  {

 while (top1 && a[st1[top1]] > = a[i]) top1—;

 while (top2 && a[st2[top2]] < = a[i]) top2—;

 if (!top1) Rs[i] = n+1;

 else Rs[i] = st1[top1];

 if (!top2) Rb[i] = n+1;

 else Rb[i] = st2[top2];

 st1[++top1] = i;

 st2[++top2] = i;

  }

  for (int i = 1; i < = n; i++)

  {

 ans + = (i—Lb[i]) * (Rb[i]—i) * a[i];

 ans - = (i—Ls[i]) * (Rs[i]—i) * a[i];

  }

  cout << ans;

  return 0;

}

#### Optimization of probability matrix decomposition

In the probability matrix decomposition algorithm, the random gradient descent method is used to learn the characteristic matrix of users and data sequences. However, each prediction error $err=R_{ui}-H_{i}^{T}Q_{j}$ in the learning process is only scored for a single user data sequence, resulting in the random gradient descent method without considering the overall prediction error effect. No matter how to optimize the feature vectors of users and data sequences, all predictions cannot be accurate, which leads to the phenomenon that although the algorithm has good prediction ability in the training set, it has poor prediction ability in the test set. To solve this problem, an adaptive error *Perr* is introduced, which is calculated by Eq (11):

Perr=err+err×w×numRate (11)

Where: *w* is the sample weight, and the initial value conforms to the uniform distribution; *numRate* is the number of scores in the training set, which results in the minimum initial value of *w*. Therefore, in Eq (11), *w* is multiplied by *numRate* to amplify the influence of *w*. In the optimization process, when the prediction error *err* is large, the sample weight *w* will become larger, and the adaptive error *Perr* will be larger; Similarly, the smaller the values of *err* and *w* are, the smaller the value of *Perr* is. The variation of *w* makes *Perr* associated with the global error. The random gradient descent method finds the appropriate eigenvector by minimizing the test error *err* of the training set [14], which is modified to minimize the adaptive error *Perr* of the training set to find the appropriate eigenvector. Therefore, the core idea of this paper is to optimize the global error as well as the single sample error more effectively.

Here, it is worth considering how to define the judgment threshold of prediction error. When *Perr* is small to a certain extent, if it continues to learn, it will lead to over fitting of the algorithm. Similar to the classification threshold of ADA Boost, it needs to meet the global needs. It can be known that standard deviation is the most commonly used quantitative form to reflect the degree of data dispersion. If the mean *errMean* of the error is used to represent the mean of the absolute prediction error of all samples, then the standard deviation *errStd* represents the dispersion degree of the overall error of the samples, that is, the greater the value of *errStd* is, the lower the prediction accuracy is, in turn, the higher the prediction accuracy is. Suppose *φ* represents the number of scores in the training set, then *errStd* is calculated with Eq (12):

$$errStd=\sqrt{\sum_{\varphi =1}^{\varphi }{\left|err\right|}^{2}-errMean^{2}}$$ (12)

In Ada Boost, the weight of the weak classifier represents the impact of the weak classifier on the final strong classification, which is derived by optimizing an index loss [19–21]. Similarly, in order to improve the optimization speed of the algorithm, this weight is also added. If the threshold value of prediction error is *η*, then:

η=a×errSt (13)

Where: *a* represents the weight of the learner, and the weight calculation method is the same as that of ADA boost weak classifier. In Ada Boost, the error rate represents the proportion of samples with classification errors in the total samples [22, 23]. However, in the mining problem, the error rate *C* is used to represent the global error in the current learner, and *φ* is set to represent the number of scores in the training set, then Eq (14) calculates *C*:

$$C=\sum_{\varphi =1}^{\varphi }w_{ui}\times |err|$$ (14)

Therefore, according to *ε*, the sample weight *w* can be adjusted automatically to prevent over fitting to a certain extent [24, 25]. The method of adjusting the sample weight *w* is shown in Eq (15):

$$D_{t+1}(i)=\frac{1}{Z_{t}}\times D_{t}(i)\times \{\begin{array}{l} \exp(C^{t})\\ \exp(-C^{t}) \end{array}$$ (15)

$$Z_{t+1}=\sum_{i=1}^{N*M}D_{t+1}(i)$$ (16)

In Eq (15): *D*_*t*_ represents the weight distribution of the sample at the *t*-th learning; *D*_*t*_(*i*) represents the *i*-th sample weight at the *t*-th learning; *Z*_*t*_ represents the normalization factor of the *t*-th learning. In Eq (16), *Z*_*t*+1_ represents the calculation method of normalization factor at the *t*+1-th learning. *C*_*t*_ represents the feature weights of the sample at the *t*-th learning.

From the implementation process of the optimized probability matrix decomposition algorithm, it can be seen that in the *t*-th learning process, the characteristic matrices *H*_*t*_ and *Q*_*t*_ are mainly learned according to *Perr*, and then *Perr* is updated according to the value of the characteristic matrix. ADA Boost only adjusts the sample weight within each random gradient descent learning. After learning convergence, ADA Boost algorithm also ends; However, the idea of ADA Boost integration is to combine multiple probability matrix decomposition algorithms with different parameters, but does not optimize a single probability matrix decomposition model itself. This is the essential difference between ADA Boost adaptive lifting idea and ADA Boost integration idea. At the same time, according to the needs of real data, the parameters *ξ*_1_ and *ξ*_2_ are introduced: in order to ensure that the algorithm does not converge at the beginning, *ξ*_1_ is used to reduce the influence of *w*; *ξ*_2_ is to restore *w* in the learning process and strengthen the intervention of *w* in the prediction error.

Eq (17) is used to initialize the sample weight:

$$w=\frac{1}{numRate\times \xi_{1}}\times \mu$$ (17)

In Eq (17), *μ* represents the error correction coefficient.

Meanwhile, improved Eq (11) is Eq (18) to calculate *Perr*. The values of parameters *ξ*_1_ and *ξ*_2_ can be obtained from cross validation experiments.


$$Perr=err+err\times w\times numRate\times \xi_{2}$$ (18)

## Results

In order to verify the mining algorithm of accumulation sequence of unbalanced data under the probability matrix decomposition studied in this paper, the accumulation sequence of unbalanced data provided by MovieLens25M is used as the experimental data accumulation sequence. The accumulation sequence contains 9 groups of data. Among them, KDD-Cup99 Intrusion dataset has 5 samples, Mammography dataset has 2 samples, and Satimage dataset has 2 samples. The algorithm in this paper is used to mine and test them. Two DELL high-density servers are used in the experiment, and each computing node is configured as follows: CPU is 16Core; Memory is 128GB; Disk is 3TB and bandwidth is 1000Mb/s. Run KVM virtual machine. Configuration: CPU is 2core; Memory is 4GB; Disk is 50GB; Bandwidth is 1000Mb/s. The server adopts Debian 6.0.5, and the virtual machine adopts Windowsver2008 operating system.

### Preprocessing test of accumulation sequence of unbalanced data

**Fig 1:**
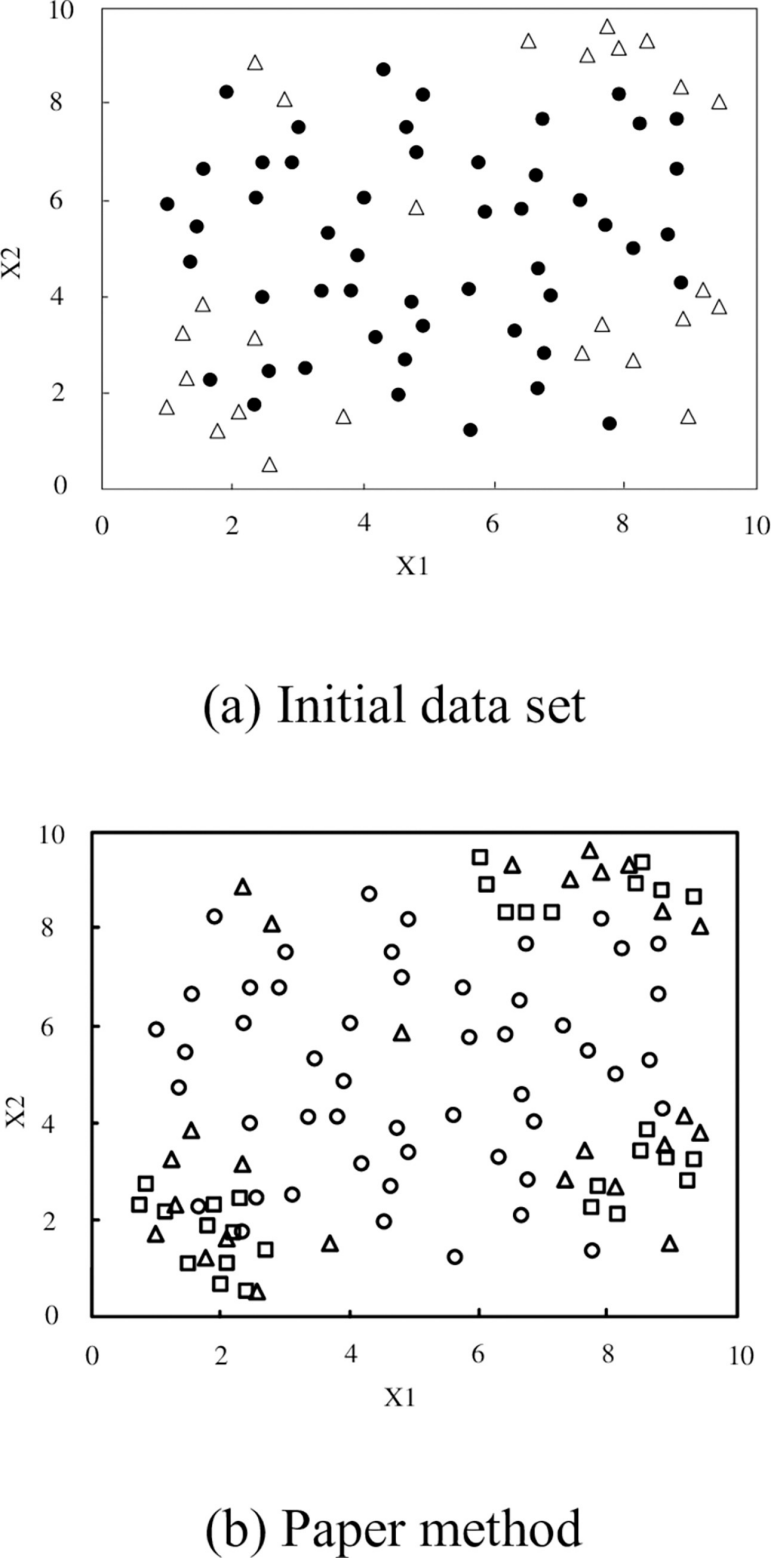
Test results of composite sample distribution. (a) Initial data set, (b) Paper method.

In order to verify the effectiveness of the proposed method, the first set of data in the experimental data accumulation sequence is used to compare the distribution of new samples generated by different oversampling methods. Suppose the data sample point is (*x*_*i*_,*y*_*i*_), where *x*_*i*_ is a two-dimensional feature, which is uniformly distributed in both dimensions, and *y*_*i*_ is a class label. The algorithm in this paper is used to process the accumulation sequence of experimental data to generate a few samples. The results are shown in Fig 1. In Fig 1, most classes are represented by a hollow circle, a few classes are hollow triangles, and the newly generated minority samples are represented by a hollow square. The number is the difference between the majority samples and the minority samples. The solid circle is most of the classes in the initial dataset, and the hollow circle is most of the classes in this method.

Fig 1(A) shows the original distribution of the data. Most classes contain a small number of noise points in minority samples; Fig 1(B) shows the distribution of new samples synthesized by the algorithm in this paper. By comparing Fig 1(A) and 1(B), it can be seen that the algorithm in this paper is used to process the accumulation sequence of experimental data, less noise points are introduced in the process of generating minority samples, and the generated new samples are more evenly distributed in the two clusters of minority classes, with less overlapping samples.

In order to further verify the effectiveness of the algorithm in this paper, the algorithm in reference [6] proposed adequacy of h-likelihood estimation method for unbalanced clustered counting data models, the algorithm in reference [7] proposed A novel hybrid feature selection and ensemble learning framework for unbalanced cancer data diagnosis with transcriptome and functional proteomic, the algorithm in reference [8] proposed A metaheuristic optimization approach for parameter estimation in arrhythmia classification from unbalanced data are taken as the comparison algorithm, which are named as comparison method 1, comparison method 2 and comparison method 3 respectively. The algorithm in this paper and the three comparison algorithms are used to cluster the eight groups of data in the accumulation sequence of the experimental data after the samples are generated. The data information of each group is shown in Table 1, in which the imbalance rate is the ratio of the number of samples in a few categories to the number of samples in a majority category. For multi-class data, one class is set as a minority class and the rest as a majority class. The eigenvalues of all sample points are scaled to [0,1]. All the data are divided into training set and test set by the method of 50% cross validation, and the average value is taken as the experimental result.

**Table 1:** Description of data sets.

Data group	Number of samples	Attribute	Multiclass	Few classes	Unbalance rate
1	21110	17	19485	1625	0.083397485
2	225	10	198	27	0.136363636
3	225	10	196	29	0.147959184
4	350	9	279	71	0.254480287
5	1560	9	1328	232	0.174698795
6	870	19	597	273	0.457286432
7	421	4	236	185	0.783898305
8	465	35	298	167	0.560402685

F-value, G-mean and AUC, the commonly used evaluation indicators for unbalanced data classification, are selected as the classification results. Where AUC is the sum of the areas of each part under the ROC curve, indicating the probability that the classifier will rank the positive instances of the random test higher than the negative instances of the random test. The larger the value is, the better the classification performance is. The calculation process of F-value and G-mean is constructed from the confusion matrix. The definition of confusion matrix is shown in Table 2.

**Table 2:** Confusion matrix.

	Predicted positive (minority) samples	Predicted as negative (majority) samples
Positive (minority) samples	Correctly classified positive number classes(TP)	Misclassified negative classes(FN)
Negative (majority) samples	Misclassified positive number classes(FP)	Correctly classified negative classes(TN)

In each index, the larger the F-value is, the better the classification performance of a few classes is. G-mean considers the classification accuracy of both majority and minority classes, and can be used to measure the overall classification effect.

Table 3 shows the F-value, G-mean and AUC values obtained by clustering with different methods. The best data are marked in black bold. For each evaluation index, the values obtained by the four unbalanced treatment methods are sorted from the best to the worst. The optimal sequence number is 1, increasing in turn. The average ranking of the four methods in all data accumulation sequences is given in the last row of the table, and the best ranking is marked in black bold.

**Table 3:** Comparison of clustering effects.

Different excavation methods	Index	Data group								Ranking
		1	2	3	4	5	6	7	8	
Comparison method 1	F-value	0.9888	0.8933	0.9765	0.9754	0.8958	0.8525	0.7902	0.9747	2.55
	G-mean	0.9695	0.6850	0.8643	0.9367	0.7878	0.8305	0.6495	0.9690	3.00
	AUC	0.9696	0.7152	**0.8823**	0.9375	0.7901	0.8339	0.6598	0.9701	2.78
Comparison method 2	F-value	0.9874	0.8855	0.9742	0.9667	0.8709	0.8545	0.7582	0.9733	3.78
	G-mean	0.9611	0.5139	0.8469	0.9284	**0.7983**	0.8443	0.6415	0.9737	3.22
	AUC	0.9613	0.6007	0.8656	0.9295	**0.7985**	0.8497	0.6478	0.9741	3.22
Comparison method 3	F-value	0.9863	0.9077	0.9742	0.9700	0.8870	0.8573	0.8239	0.9745	2.89
	G-mean	**0.9701**	0.5233	0.8469	0.9317	0.7885	0.8431	0.6501	**0.9749**	**2.44**
	AUC	**0.9701**	0.6290	0.8656	0.9324	0.7899	0.8477	0.6692	**0.9753**	**2.55**
Algorithm in this paper	F-value	**0.9944**	**0.9088**	**0.9785**	**0.9813**	**0.9092**	**0.9063**	**0.8242**	**0.9763**	**1.11**
	G-mean	0.9674	**0.6959**	**0.8662**	**0.9421**	0.7892	**0.8612**	**0.6628**	0.9724	1.67
	AUC	0.9678	**0.7173**	0.8676	**0.9432**	0.7938	**0.8626**	**0.6826**	0.9725	1.78

The comparison results in Table 3 show that:

1. Average ranking. Compared with the algorithms in reference [6], reference [7] and reference [8], the algorithm in this paper has better classification performance on the accumulated sequence of balanced data processed by the algorithm, and the average ranking of indicators F-value, G-mean and AUC are the best, which proves that the algorithm in this paper has obvious advantages in processing unbalanced data.
2. F-value. The algorithm in this paper achieves the optimal value on all data sets. The three comparison methods achieve the optimal value in a few data sets. Overall, this algorithm effectively improves the classification accuracy of a few samples.
3. G-mean and AUC values. In the classification results, the proposed algorithm achieves the optimal value in most data accumulation sequences, which proves that the proposed method improves the overall classification performance of unbalanced data.
4. On data group No. 2, No. 4, No. 6 and No. 7, the algorithm in this paper obtains the optimal F-value, G-mean and AUC values at the same time.
5. The reason is that this algorithm takes into account the imbalance problem in a few samples, while other methods do not take into account the internal distribution of samples.

### Analysis of excavation performance

The accuracy of mining performance is used to measure the consistency between the mining results calculated by the algorithm in this paper and the real results given by users. The error is used to indicate whether the mining results meet the needs of users. In this paper, RMSE is selected as the evaluation index to analyze the impact of different conditions on the mining performance of this method.

#### Influence of parameter setting on mining performance of the proposed algorithm

**Fig 2:**
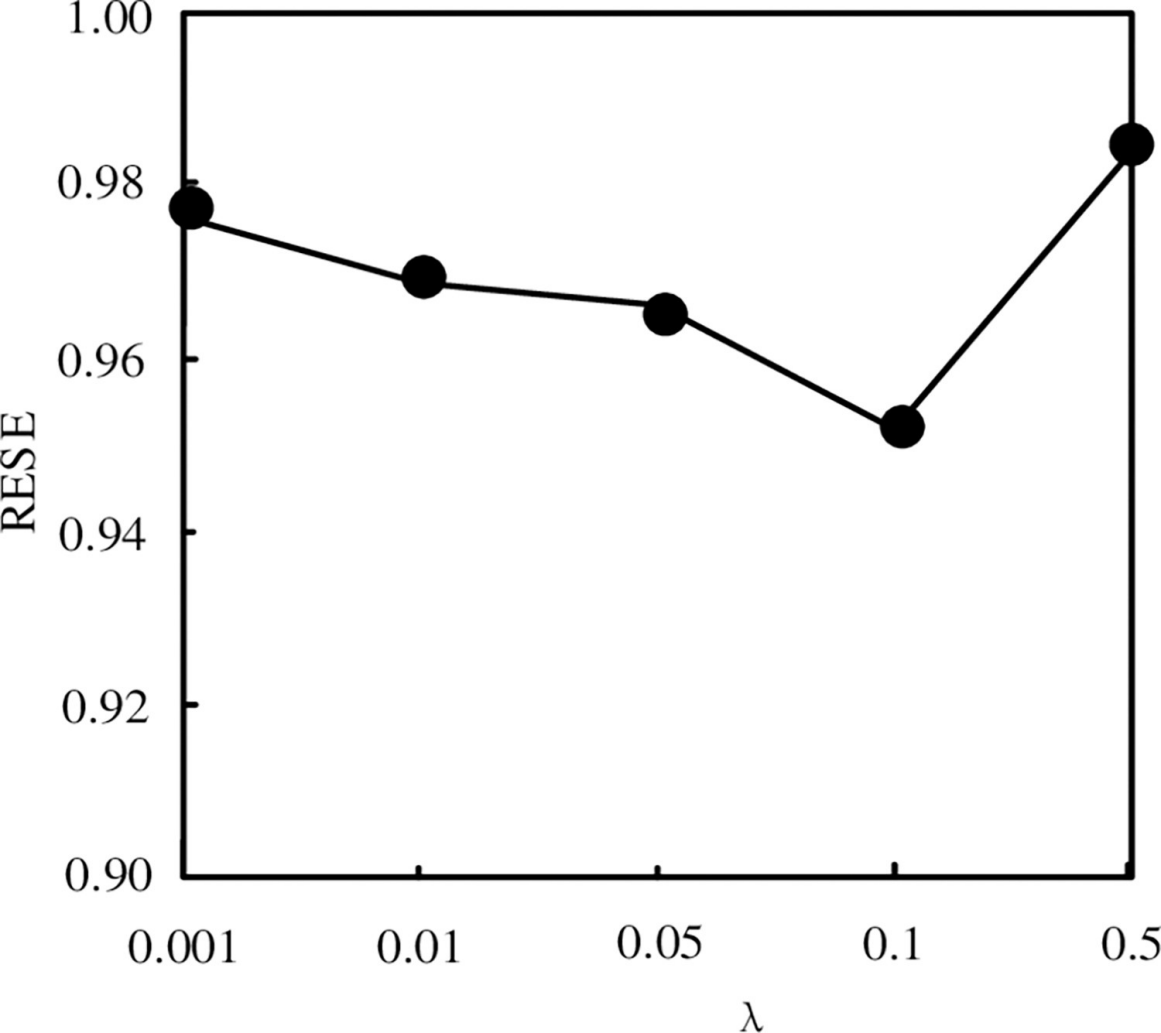
Influence of parameter setting on mining performance.

According to Eq (7), $\sigma_{R}^{2}$ and $\sigma_{H}^{2}$, $\sigma_{H}^{2}$ and $\sigma_{Q}^{2}$, where $\sigma_{R}^{2}$ is the variance of Gaussian observation noise $\sigma_{H}^{2}$ = $\sigma_{Q}^{2}$. The cross validation method and empirical parameters of $\sigma_{R}^{2}$ can be obtained according to the experimental data. In the experiment, the influence of *λ* = 0 = 0.01, 0.01, 0.05, 0.1, 0.5 on RMSE is dynamically considered. During the experiment, the decomposition dimension is taken as 5 and the result is iterated for 50 times. The experimental results are shown in Fig 2.

It can be seen from Fig 2 that when *λ*∈[0.05,0.1], the square root error of the model is obviously lower. In the following experiments, *λ* = 0.1 is taken as the optimal value.

#### Impact of decomposition dimension and iteration times on mining performance

**Fig 3:**
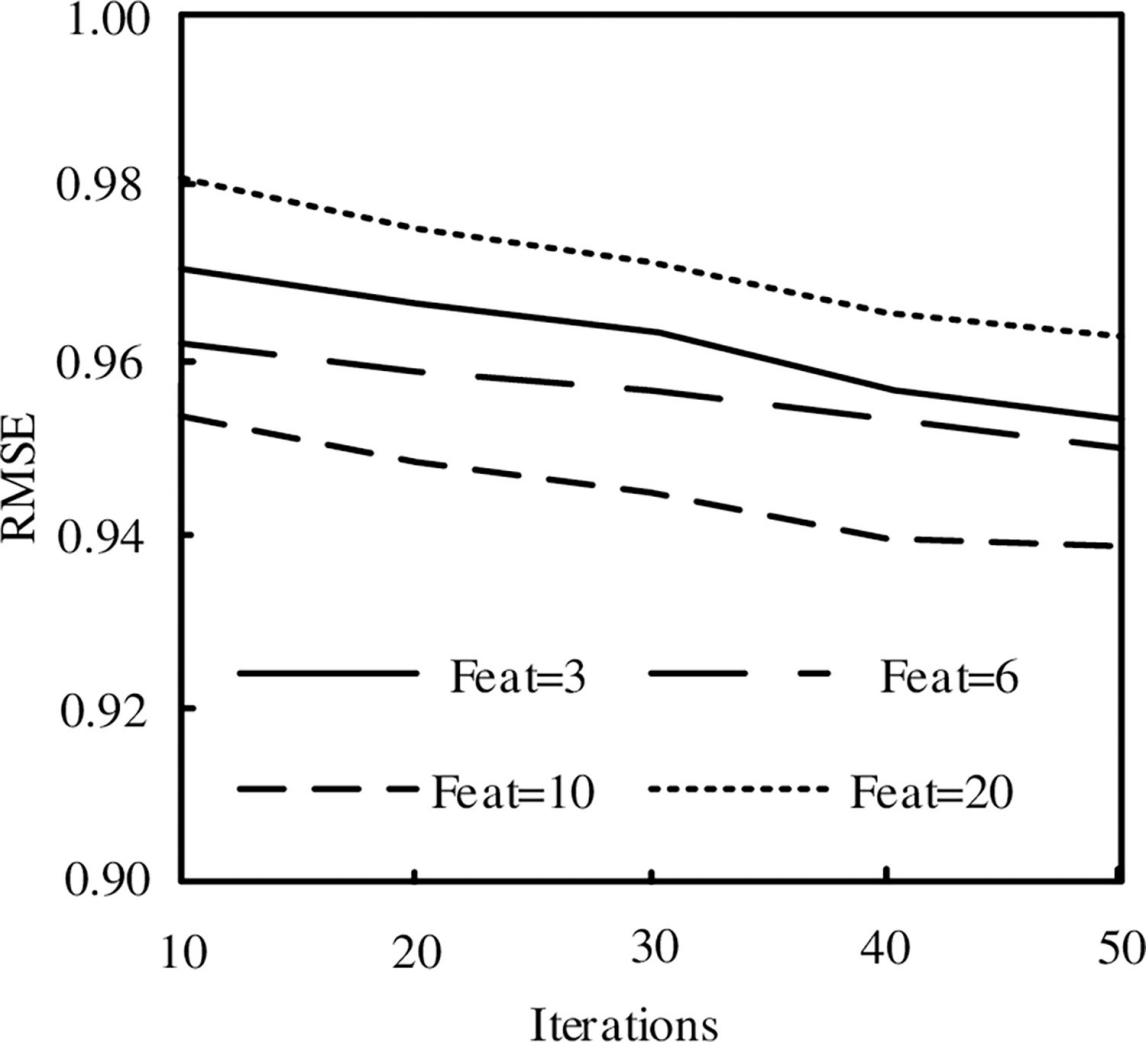
Impact of decomposition dimension and iteration times on mining performance.

In the experiment, the influence of decomposition dimension and iteration times is dynamically considered. The iteration times are 10, 20, 30, 40 and 50 respectively, and the decomposition dimensions are 3, 6, 10 and 20. The experimental results are shown in Fig 3.

As can be seen from Fig 3, when the decomposition dimension is unchanged, RMSE decreases slightly with the increase of iteration times; When the number of iterations does not change, the decomposition dimension first decreases and then increases. When the decomposition dimension is, 20, the minimum RMSE is obtained. Therefore, the number of iterations is 50, and when the decomposition dimension is 5, the minimum RMSE is obtained.

#### Analysis of mining accuracy

In order to further verify the mining effect of the proposed method, it is compared with the method in literature [6] and the method in literature [7] to test the mining accuracy, and the comparison results are shown in Table 4.

**Table 4:** Comparison of mining accuracy.

Number of iterations	Textual method	Method of literature [6]	Method of literature [7]
1	0.941	0.814	0.831
2	0.931	0.815	0.831
3	0.930	0.812	0.835
4	0.929	0.810	0.837
5	0.923	0.818	0.837
6	0.985	0.817	0.828
7	0.993	0.816	0.825
8	0.950	0.812	0.820
9	0.938	0.811	0.827
10	0.928	0.802	0.826
11	0.981	0.801	0.822

According to the analysis of Table 4, the mining accuracy of the method in this paper is above 0.92, and that of the two literature methods is below 0.84. Compared with the two literature methods, the mining accuracy of the method in this paper is higher.

## Conclusion

With the advent of the information age, the generation of data is increasing. Although such a huge data system can provide enough information for decision-making, it also poses new challenges for the application of these large-scale data. The application of accumulation sequence of unbalanced data is one of the difficult problems in data application, which is mainly caused by the characteristics of accumulation sequence of unbalanced data. To solve these problems, this paper studies the mining algorithm of accumulation sequence of unbalanced data based on probability matrix decomposition. The accumulation sequence of unbalanced data is balanced, and then the accumulation sequence of balanced data is mined by probability matrix decomposition. Aiming at the problem that the overall prediction error effect is not considered in the traditional probability matrix decomposition process, the adaptive error is introduced to optimize it, and the mining results are obtained.

Only by deeply understanding the essence of data accumulation sequence can this paper design a more suitable algorithm to deal with this problem. At present, the research on the mining of accumulation sequence of unbalanced data is still in its infancy, and there is still a lot of room for development, which also puts forward many open challenges for future research.
